# An Individualized, Perception-Based Protocol to Investigate Human Physiological Responses to Cooling

**DOI:** 10.3389/fphys.2018.00195

**Published:** 2018-03-13

**Authors:** Crystal L. Coolbaugh, Emily C. Bush, Elizabeth S. Galenti, E. Brian Welch, Theodore F. Towse

**Affiliations:** ^1^Vanderbilt University Institute of Imaging Science, Vanderbilt University Medical Center, Nashville, TN, United States; ^2^Department of Radiology and Radiological Sciences, Vanderbilt University Medical Center, Nashville, TN, United States; ^3^Department of Biomedical Engineering, Vanderbilt University, Nashville, TN, United States; ^4^Department of Biomedical Sciences, Grand Valley State University, Allendale, MI, United States

**Keywords:** thermoregulation, cold exposure, brown adipose tissue, supraclavicular skin temperature, vasoconstriction, shivering, humans

## Abstract

Cold exposure, a known stimulant of the thermogenic effects of brown adipose tissue (BAT), is the most widely used method to study BAT physiology in adult humans. Recently, individualized cooling has been recommended to standardize the physiological cold stress applied across participants, but critical experimental details remain unclear. The purpose of this work was to develop a detailed methodology for an individualized, perception-based protocol to investigate human physiological responses to cooling. Participants were wrapped in two water-circulating blankets and fitted with skin temperature probes to estimate BAT activity and peripheral vasoconstriction. We created a thermoesthesia graphical user interface (tGUI) to continuously record the subject's perception of cooling and shivering status during the cooling protocol. The protocol began with a 15 min thermoneutral phase followed by a series of 10 min cooling phases and concluded when sustained shivering (>1 min duration) occurred. Researchers used perception of cooling feedback (tGUI ratings) to manually adjust and personalize the water temperature at each cooling phase. Blanket water temperatures were recorded continuously during the protocol. Twelve volunteers (ages: 26.2 ± 1.4 years; 25% female) completed a feasibility study to evaluate the proposed protocol. Water temperature, perception of cooling, and shivering varied considerably across participants in response to cooling. Mean clavicle skin temperature, a surrogate measure of BAT activity, decreased (−0.99°C, 95% CI: −1.7 to −0.25°C, *P* = 0.16) after the cooling protocol, but an increase in supraclavicular skin temperature was observed in 4 participants. A strong positive correlation was also found between thermoesthesia and peripheral vasoconstriction (ρ = 0.84, *P* < 0.001). The proposed individualized, perception-based protocol therefore has potential to investigate the physiological responses to cold stress applied across populations with varying age, sex, body composition, and cold sensitivity characteristics.

## Introduction

In the decade since its rediscovery in adult humans (Nedergaard et al., 2007), brown adipose tissue (BAT) has emerged as a target of intense physiological and therapeutic interest. Cold exposure, an established stimulant of the thermogenic effects of BAT, is the most widely used method for understanding BAT physiology (Cypess et al., 2009; van Marken Lichtenbelt et al., 2009; Virtanen et al., 2009; Ouellet et al., 2012). Limited consensus, however, exists on how to apply the cold stimulus to ensure comparable results across research participants with varying physiological characteristics or pathological conditions (Chen et al., 2016). Recently, individualized, water-cooling protocols have been recommended to standardize physiological cold stress and increase the likelihood of activating BAT equally across participants [see review of BAT cooling protocols (van der Lans et al., 2014)]. Experimental details, however, remain unresolved: how should cooling start and progress? what is an appropriate physiological marker of cold stress?

Recent studies reveal a variety of experimental practices and analytical approaches to address these questions. Water temperatures may start near skin temperature (between 31 and 35 °C) or at distinct temperatures (as cold as 8°C) (Lee et al., 2014; Salem et al., 2016; Martinez-Tellez et al., 2017a,b). Coolant temperatures may then change in an undefined manner (Vijgen et al., 2011; Vosselman et al., 2012), move in a single step and remain fixed (Ouellet et al., 2012; Muzik et al., 2013; Matsuda-Nakamura et al., 2015; Gifford et al., 2016), or shift along a gradient with small (1°C) or large steps (5°C) over varied time periods (e.g., 5–30 min) (van der Lans et al., 2013; Bakker et al., 2014; Chondronikola et al., 2016; Martinez-Tellez et al., 2017a,b). Cooling then typically terminates at the onset of shivering—a common physiological event used to indicate equal cold stress across participants. Approaches to define and measure the onset of shivering, however, often differ between research groups (van der Lans et al., 2014). Collectively, these subtle inconsistencies in cooling protocols may fail to induce cold exposure equally and complicate efforts to compare results among research groups. Standardized protocols are needed to ensure that differences in the prevalence and thermogenic properties—including but not limited to the timing and magnitude of heat production - of BAT among participants are a true physiological response of the tissue to cold exposure and not a result of methodological variability (van der Lans et al., 2014; Chen et al., 2016).

Here, we propose an individualized, perception-based protocol to investigate human physiological responses to cooling, with a special interest in BAT. In accordance with recent efforts to standardize and validate methods to study BAT physiology (Chen et al., 2016), we provide experimental details and analytical tools to guide researchers from subject preparation through data analysis. A feasibility study was conducted to assess the efficacy of the protocol and to explore the use of thermoesthesia (the ability to detect changes in temperature) to guide the progression of cooling. We hypothesize that changes in thermoesthesia relate to cold-induced responses in peripheral vasoconstriction. If so, thermal sensation feedback may allow researchers to adapt the cooling protocol according to the participant's cold sensitivity - an important consideration to replicate cold stress during longitudinal or cold acclimatization intervention studies of BAT physiology (Chen et al., 2016).

## Materials and methods

### Design criteria for an individualized, perception-based cooling protocol

In designing this protocol, we considered the following criteria:

- Achieves a similar cold-induced physiological response across participants.
- Adapts cooling according to the participant's cold sensitivity.
- Quantifies the physiological response to cold exposure (e.g., perception of cooling, skin temperature gradients, and shivering).

### Delivering the cooling stimulus

**Figure 1:**
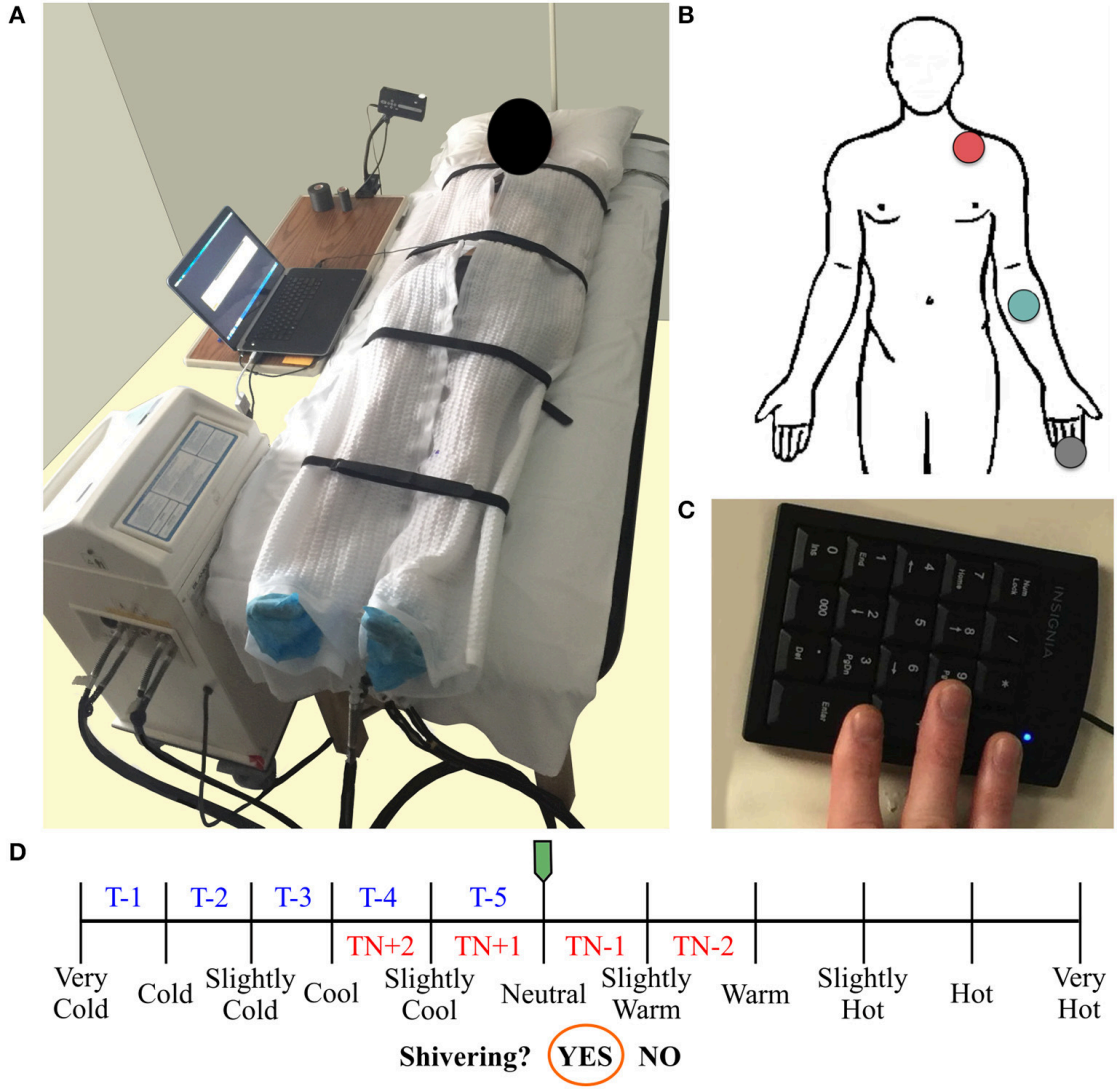
Participant's setup used for an individualized, perception-based cooling protocol **(A)**. Two water-circulating blankets connected to a Blanketrol® III hyper-hypothermia system are secured around the participant. Diagram of the skin temperature probe locations (human body outline adapted from a free graphics library: http://clipart-library.com) **(B)**. Skin temperature was measured in the supraclavicular region (red circle), on the anterior portion of the forearm (green circle), and on the distal end of the middle finger (gray circle). A laptop computer logs water temperature and runs a thermoesthesia graphical user interface (tGUI) program. The tGUI records feedback from an external keypad **(C)** attached to the participant's right hand (positioned underneath the blankets during cooling) and updates a visual display projected above the patient bed in real-time. Schematic of the perception-based cooling protocol water temperature adjustment guide (blue and red text) overlay on the tGUI visual interface **(D)**. During the protocol, the participant uses two buttons to move the green slider along the continuum of temperature ratings. The slider's position determines how to alter the current water temperature set point (TN during thermoneutral (red text) or T during cooling (blue text) phases; see section Individualized, Perception-Based Cooling Protocol for details). A third button can be pressed to move the orange circle and indicate if shivering is active (“Yes”) or inactive (“No”).

We used a Blanketrol® III hyper-hypothermia system (Cincinnati Sub-Zero, Cincinnati, OH, USA) to cool the participant (Figure 1A). Prior to the start of a session, two water-circulating blankets (Maxi-Therm® Lite Model, Cincinnati Sub-Zero, Cincinnati, OH, USA) were taped together to prevent overlap of the cooling area and to secure their position on top of a patient bed. The blanket water temperature was then warmed to 32°C (a preliminary estimate of thermoneutral). The participant laid in a supine position on top of the blankets such that the blanket's top edge was aligned with the base of the chin. Straps secured the blankets around the participant's body with care taken to keep the participant's arms and legs slightly abducted to limit skin contact between body parts and to cover as much of the participant's body as possible. Water temperature was adjusted with the Blanketrol® III interface according to the protocol guidelines. Set and achieved water temperatures were recorded via a laptop computer (Blanketrol® III Data Export Software) every 30 s.

### Monitoring acute physiological responses to cold exposure

#### Skin temperature

Changes in supraclavicular skin temperature have been shown to relate positively with BAT volume and activity in response to cold exposure (Boon et al., 2014; Chondronikola et al., 2016; van der Lans et al., 2016). Additional skin temperature measurements and calculated gradients can also be implemented to monitor physiological responses to cooling (Martinez-Tellez et al., 2017a). Here, we describe a minimal set of skin temperature measurements needed to estimate BAT activity (Boon et al., 2014; Chondronikola et al., 2016; van der Lans et al., 2016) and peripheral vasoconstriction (Rubinstein and Sessler, 1990). Three temperature probes (Model MLT422/A; ADInstruments, Colorado Springs, CO, USA) were placed above the clavicle (superficial to a potential BAT depot), on the anterior portion of the forearm, and on the distal end of the middle finger (Figure 1B). The arm-to-finger skin temperature gradient was calculated to reflect peripheral vasoconstriction (Rubinstein and Sessler, 1990). Probes were secured to the skin with adhesive tape and covered with a thin cotton pad to avoid direct contact with the cooling blanket. Temperature signals were sampled every 30 s, and analyzed with LabChart software (Thermistor Pod Model ML309, PowerLab 16/35 Data Acquisition System, LabChart version 8; ADInstruments, Colorado Springs, CO, USA).

#### Perception of cooling (thermoesthesia)

We developed a thermoesthesia Graphical User Interface (tGUI) (Welch et al., 2017) to allow participants to evaluate differences in temperature and shivering in real-time. The tGUI tool is freely available for download (https://github.com/welcheb/Thermoesthesia_GUI) and can be executed in MATLAB (Mathworks, Natick, MA, USA). The tGUI visual interface consists of a series of qualitative descriptors marked on a temperature continuum and a binary shivering event capture (Figure 1D). Each position on the temperature scale registers a numeric value ranging from 0 (“Very Cold”) to 100 (“Very Hot”) in single integer increments. During a cooling session, a small projector displayed the tGUI visual analog scale interface above the patient bed. The participant's right hand was attached to a small, numeric keypad (Figure 1C), which rested next to the participant's right leg underneath the cooling blankets. The participant used two buttons on the keypad to position a slider along the continuum of temperature ratings and a third button to indicate if shivering was active (“Yes”) or inactive (“No”). The tGUI tool recorded the current date and time, the numeric slider value, and the current status of the shiver indicator (No = 0; Yes = 1) every 30 s and each time a key was pressed.

#### Shivering

Surface electromyography (EMG), self-report (tGUI tool), and observation were combined to record shivering events during the cooling protocol. Bipolar surface electrodes (Octal Bio Amp Model FE238, Powerlab 16/35 Data Acquisition System; ADInstruments, Colorado Springs, CO, USA) were placed on the belly of the trapezius (TRAP) and sternocleidomastoid (SCM)—superficial muscles known to contribute to shivering upon cold exposure (Tikuisis et al., 1991; Bell et al., 1992). The reference electrode was placed on the distal end of the humerus on the same side of the body as the other electrodes, and participants performed a series of isometric contractions to confirm recording of muscle activity. Throughout the cooling session, participants self-reported shiver events with the tGUI tool (section Perception of Cooling (Thermoesthesia)), and members of the research team noted the start and end time of any body movements (e.g., shiver, yawn, speaking, fidget, etc.) as a reference standard for any EMG muscle activity.

##### EMG shiver event detection analysis

Raw EMG signals were sampled at 1,000 Hz, band-pass analog filtered 20–400 Hz, and notch filtered at 60 Hz to remove motion artifacts and electrical noise, respectively, using LabChart software. Custom MATLAB scripts were then used to detect shivering events in the processed EMG data. In brief, a Teager-Kaiser energy operator transformed the EMG signal to improve the signal to noise ratio (Li et al., 2007; Solnik et al., 2010). The Teager-Kaiser signal was rectified and low-pass filtered at 50 Hz, and a portion of the signal was selected when the muscle was at rest (approximately 5 s of data) to calculate a baseline reference value. The threshold crossing detection (Li et al., 2007; Solnik et al., 2010) used to identify the onset of muscle activity was defined by

(1) $$\begin{array}{r} y_{Threshold}=\;\mu +J•\sigma \end{array}$$

where μ and σ equal the mean and standard deviation of the baseline reference and J is 15, a preset value validated by Solnik et al. (2010) for low magnitude muscle activity baselines. Shiver onset was recorded as the first time point to exceed and stay above the threshold for at least 10% of a 250 ms sliding window. Shiver events were combined if less than a 50 ms gap existed between the off and on times of neighboring events.

### General participant preparation guidelines

Numerous factors, including age, sex, body composition, diet, cold acclimatization, and exertional fatigue, contribute to individual variability in the physiological response to cold (Castellani and Young, 2016); therefore, participant characteristics were documented and pretest conditions were controlled for the perception-based cooling protocol (Chen et al., 2016). Exclusion criteria included: habitual tobacco or alcohol use, significant changes in body weight (>5% in the past 3 months), use of prescribed and over-the-counter medications known to affect thermoregulation (Cuddy, 2004; Mukherjee et al., 2016), breast feeding, and pregnancy (Chen et al., 2016). Prior to the test session, participants consumed no food or beverage other than water for a minimum of 6 h to limit the effect of diet and caffeine on BAT activity (Vosselman et al., 2013; Mukherjee et al., 2016). Participants also avoided vigorous (relative intensity > 7 using a scale of 0 (none) to 10 (maximal effort)) and moderate (relative intensity between 5 and 7) physical activity for 72 and 24 h before the session, respectively (US Department of Health and Human Services, 2008). To limit the effects of sex hormones on thermoregulation (Charkoudian et al., 1999; Charkoudian and Stachenfeld, 2016), female participants completed testing during the early follicular phase (days 1–4) of the menstrual cycle. Repeat cooling protocols were also scheduled at the same time of day, preferably in the morning, to limit diurnal differences in skin blood flow (Aoki et al., 2003). Finally, male and female participants wore lightweight clothing with minimal skin coverage to enhance heat transfer with the water-circulating blankets (e.g., male: briefs 0.04 clo, shorts 0.06 clo, socks 0.02 clo; female: underwear 0.03 clo, bra 0.01 clo, shorts 0.06 clo, socks 0.02 clo). Clo units refer to clothing insulation values (Bligh and Johnson, 1973; American Society of Heating Refrigerating and Air-Conditioning Engineers, 2005).

### Familiarization session

We required participants to complete a familiarization session at least 2 days and no longer than 30 days prior to a perception-based cooling protocol. The purpose of this session was to introduce the participant to the cooling environment, the sensation of cold exposure and shivering, and use of the tGUI tool. Although it was not necessary to adhere to pretest restrictions for the familiarization session, participants wore appropriate clothing (section General Participant Preparation Guidelines), and skin temperature sensors were used to confirm cooling. Water temperature adjustments for the familiarization session (Table 1) exposed the participant to a wide range (32°-7°C) and large decrements (−10°C) in cooling in a short period of time to allow for ample training with the tGUI tool. Careful instruction regarding the use of the tGUI tool was provided to the participant throughout the session to encourage careful evaluation of thermoesthesia and to express the occurrence of shivering events in a standard format (Table 2). The familiarization session ended either when the participant reported sustained shivering for longer than 1 min or at the end of the seventh stage (8 min at 7°C) of the cooling paradigm.

**Table 1:** Familiarization session water temperature adjustment guide.

**Time (min)**	**Set Temperature (°C)**
0–8	32
8–16	22
16–24	19
24–32	16
32–40	13
40–48	10
48–56	7

**Table 2:** Recommended instructions for thermoesthesia graphical user interface (tGUI) Training.

**Training Topic**	**Recommended Instruction**
**ORIENTATION to tGUI TEMPERATURE SCALE**	
Define “Neutral”	Temperature is comfortable–not warm or cool.
Define “Very Cold”	Temperature is difficult to tolerate (coldest ever experienced).
**HOW TO USE tGUI TEMPERATURE SLIDER**	
Position	Two buttons move the slider left and right. Any position on the scale logs a value. It is not necessary to align with qualitative cues.
Timing	Update the slider position at your discretion. If water temperature is changed, wait approximately 30 s to allow flow to stabilize.
**ORIENTATION TO tGUI SHIVER EVENT LOG**	
Define “Shiver”	An involuntary muscle movement (does not include piloerection or skin sensations).
Whole-Body Awareness	Record shivers that occur anywhere in the body. Shivering may begin in core muscles (abdominals) and progress to extremities (hands, neck), but each person's experience is unique.
Timing	Hit button (“Yes”) at the start of a shiver and hit button a second time (“No”) at the end of the shiver. Shiver can be brief or longer duration.
**ERROR REPORTING**	
Errant Button Press	Report verbally to research team at time of error.

### Individualized, perception-based cooling protocol

The primary goal of the individualized, perception-based cooling protocol session was to identify the water temperature that elicited sustained shivering in a participant. This reference water temperature can then be adjusted (e.g., + 3°C) to minimize shivering if the experimental design requires continued cold exposure on the same experimental day (Chen et al., 2016) or as part of a separate session (Martinez-Tellez et al., 2017b). Prior to the start of the protocol, the water-cooling system, participant, and physiological sensors were prepared (sections Delivering the Cooling Stimulus–General Participant Preparation Guidelines) and indoor and outdoor temperature and humidity were recorded. The protocol began with a thermoneutral phase (15 min) followed by a series of cooling phases (10 min) and concluded when the participant reported sustained shivering (the tGUI shiver event indicator in the “Yes” position for longer than 1 min). For each perception-based cooling protocol, we recorded: time at start of a phase (when water flow begins), time at end of a phase (when water flow stops), thermoneutral water temperature, and shivering water temperature.

The participant's thermal perception feedback—the position of the tGUI slider on the temperature continuum—was used to guide water temperature adjustments during the protocol (Figure 1D). The protocol started at a water temperature of 32°C with the tGUI slider aligned with “Neutral.” If the participant moved the tGUI slider to a warmer or cooler position, researchers adjusted the water temperature (3 min between adjustment) until the participant returned to a “Neutral” perception, which initiated the 15-min thermoneutral phase (Figure 1D: red text = thermoneutral phase water temperature guide). At the end of the thermoneutral phase and each subsequent cooling phase, researchers evaluated the position of the tGUI slider to determine how much to decrease the water temperature set point for the next protocol phase (Figure 1D: blue text = cooling phase water temperature guide). For example, a tGUI slider position between “Very Cold” and “Cold” at the end of a phase resulted in a 1°C decrease in water temperature from the previous set point. If the slider was aligned with a qualitative landmark, the smaller decrease in temperature was used (e.g., alignment with “Cool” would result in a 3°C drop in water temperature).

### Summarizing cooling protocol data

We created an open-source MATLAB program (https://github.com/ccoolbaugh/Individualized_Cooling_Data_Analysis) to summarize raw data acquired during a perception-based cooling protocol (Coolbaugh and Bush, 2017). The program imports log files from the Blanketrol® III, tGUI tool, and skin temperature probes; synchronizes the file time stamps (start of cooling equals time zero); and generates plots of the data.

### Feasibility assessment of the individualized, perception-based cooling protocol

#### Participant characteristics

Fifteen volunteers (four females) were recruited from Vanderbilt University and the greater Nashville area using word-of-mouth and email advertisements. Volunteers were between 18 and 35 years of age, non-smokers, and apparently healthy: with no history or symptoms of cardiovascular, pulmonary, metabolic, or neurological disease and no current use of prescribed or over-the-counter medications known to affect thermoregulation (Cuddy, 2004; Mukherjee et al., 2016) as self-reported on a medical history questionnaire. Female participants were not pregnant or breast-feeding and were studied during the early follicular phase (day 1–4) of the menstrual cycle.

Volunteers reported to the laboratory on two separate occasions to complete a familiarization session and an individualized, perception-based cooling protocol (sections Familiarization Session–Individualized, Perception-Based Cooling Protocol). Prior to the perception-based cooling protocol, compliance with pre-test restrictions (section General Participant Preparation Guidelines) was assessed by a questionnaire. Participants changed into standard clothing, and body mass and height without shoes were measured using a calibrated scale and stadiometer, respectively. These values were used to calculate body mass index (BMI) (mass (kg) divided by height squared (m)). In addition, waist circumference was measured just above the ilium of the pelvis with a Gulick spring-loaded handle (Centers for Disease Control and Prevention, 2007). The Vanderbilt University Medical Center Institutional Review Board approved the experimental protocol, and all participants provided written, informed consent.

#### Statistical analysis

Three analyses were completed to evaluate the efficacy of the proposed cooling protocol to: (1) individualize cooling, (2) titrate changes in water temperature according to thermoesthesia, and (3) quantify physiological responses (i.e., perception, skin temperature, and shivering) to cold exposure. First, water temperature and the participant's perception of cooling were extracted at three physiological markers: vasoconstriction index (arm-to-finger skin temperature difference equal to 4°C, Rubinstein and Sessler, 1990), onset of shivering (first recorded tGUI shiver event), and the end of the protocol (sustained shivering longer than 1 min) to explore temporal changes in an individual's response to cooling. Linear mixed-effects repeated measures analysis was performed (R package *nlme*, Pinheiro et al., 2017) to test differences in water temperature and perception at each physiological marker. Tukey's *post hoc* pairwise comparison was applied when indicated (R package *multcomp* Hothorn et al., 2008). Second, Spearman rank correlation coefficients (ρ) were calculated to test the relationship between skin temperature and thermoesthesia ratings. Changes in clavicle, arm, and finger temperatures were expressed relative to the skin temperature of the respective anatomical location at the end of the thermoneutral phase, and the arm-to-finger temperature gradient was calculated as a measure of peripheral vasoconstriction (Rubinstein and Sessler, 1990). Paired samples Wilcoxon signed rank tests were performed to compare pre- (end of thermoneutral phase) and post- (end of protocol) alterations in relative skin temperatures. To prepare data for these analyses, perception ratings and temperatures were linearly interpolated to a time vector common to both data series and averaged across subjects. Third, the utility of EMG (combined TRAP and SCM muscle activity) and the tGUI tool to measure shivering was evaluated. EMG and tGUI shiver event times were compared, and similarity fractions (Equations 2 and 3, respectively) were calculated

(2) $$EMG_{similarity\;fraction}=\frac{EMG_{shiver}\cap tGUI_{shiver}}{\sum EMG_{shiver}}$$

(3) $$tGUI_{similarity\;fraction}=\frac{EMG_{shiver}\cap tGUI_{shiver}}{\sum tGUI_{shiver}}$$

where *EMG*_*shiver*_ and *tGUI*_*shiver*_ represent the number of EMG and tGUI shiver events, respectively. Normality assumptions were evaluated using visual inspection of histogram and quantile-quantile plots of the data. The level of significance for all statistical tests was set at *P* < 0.05, and statistical analyses were performed in R Studio (version 1.0.153; R Studio, Boston, MA, USA). Data are presented as means ± 95% confidence intervals, unless otherwise noted.

## Results

### Subject characteristics and environmental conditions

Of the 15 participants enrolled, 12 completed the feasibility study (Table 3). One male withdrew due to discomfort with cold exposure, and one female did not complete the perception-based cooling protocol during the early follicular phase of the menstrual cycle. In addition, testing for one male was canceled following an operator malfunction of the Blanketrol® III system. Participants completed both sessions in 30 days, and all testing was done at the Vanderbilt University Medical Center in Nashville, TN between December 2016 and June 2017.

**Table 3:** Participant characteristics and environmental conditions.

**Parameter**	***n* = 12 (9 male; 3 female)**
Age (years)	26.2 ± 1.4 (19 to 34)
Height (cm)	171.0 ± 2.8 (161.3 to 195.6)
Mass (kg)	69.5 ± 3.4 (54.9 to 91.6)
BMI (kg/m^2^)	23.2 ± 0.7 (19.3 to 27.7)
WC (cm)	81.4 ± 2.3 (69.3 to 97.2)
Outdoor Temperature (°C)	9.1 ± 3.0 (−6 to 26)
Outdoor Humidity (%)	74.1 ± 4.7 (48 to 100)
Indoor Temperature (°C)	21.3 ± 0.6 (17 to 24)
Indoor Humidity (%)	38.8 ± 5.5 (7 to 70)

*Values are Means ± SE (Minimum to Maximum). Environmental conditions were recorded at the start of the perception-based cooling protocol sessions only. BMI, body mass index; WC, waist circumference*.

### Individual variability of cold-induced physiological responses

**Figure 2:**
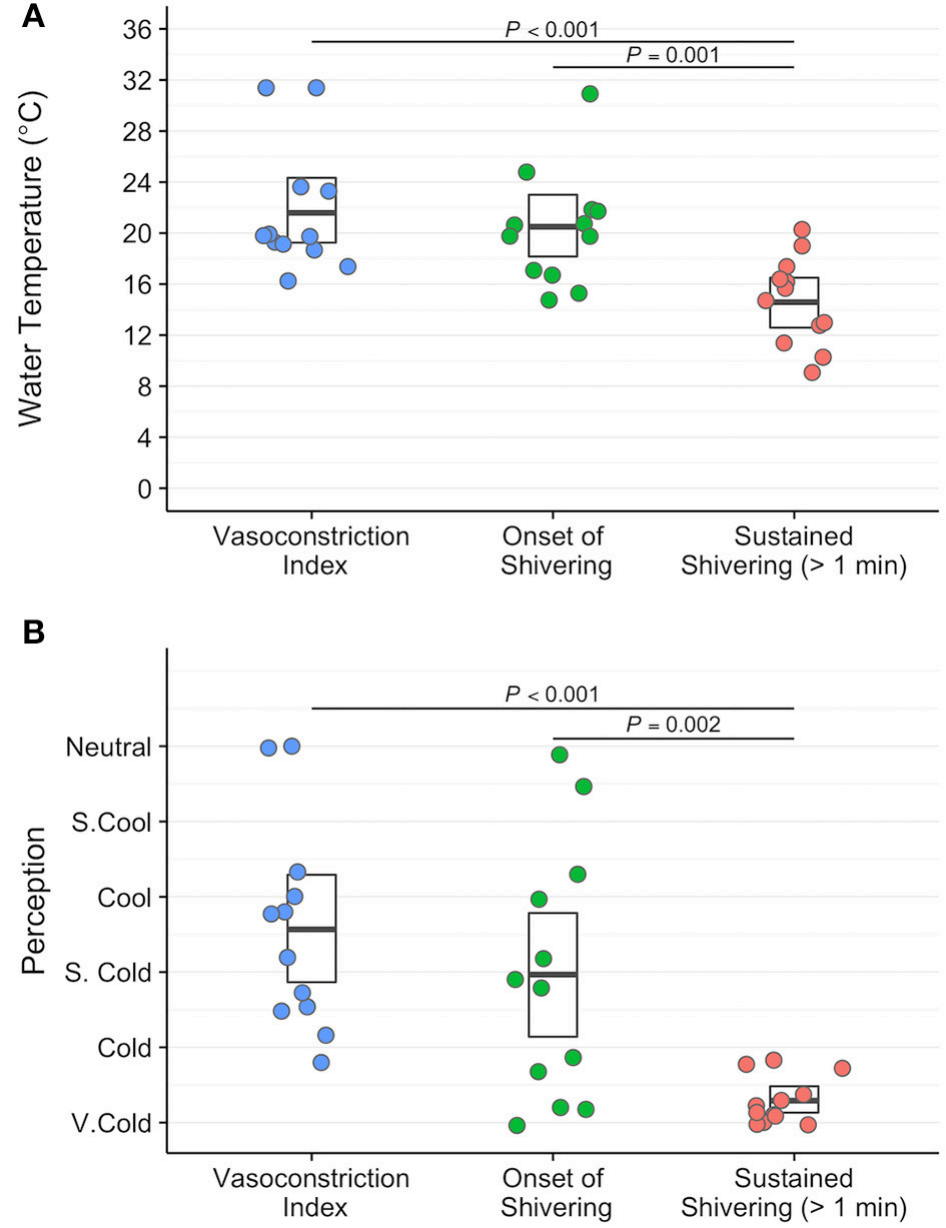
Water temperature (°C; **A**) and subjective perception of cooling ratings (arbitrary units; **B**) exhibit a wide range of variability between subjects at three physiological and temporally distinct markers: vasoconstriction index (>4°C gradient between forearm and finger skin temperature; blue circles), onset of shivering (first self-reported shiver event; green circles), and sustained shivering (self-reported shiver event with a duration >1 min; red circles) in the perception-based cooling protocol. The centerline in each box indicates the mean value, and the top and bottom of the box mark the 95% confidence intervals. Tukey's *post-hoc* pairwise tests were performed following a linear mixed-effects analysis with repeated measures to assess statistical comparisons.

**Figure 3:**
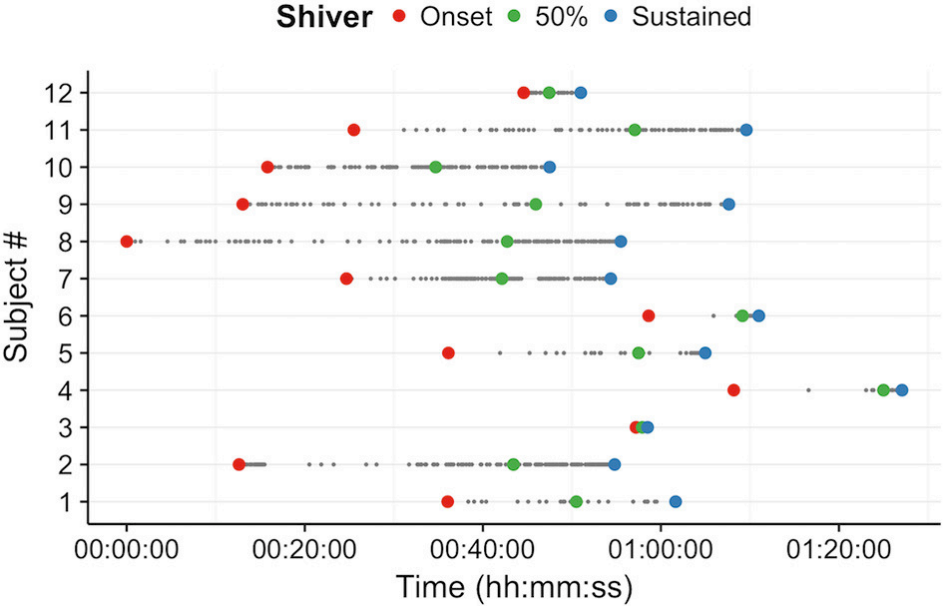
Self-reported shiver patterns (onset (red), 50% of total shiver events (green), and sustained shivering (>1 min duration; blue)) differed across participants during the individualized, perception-based cooling protocol.

Water temperature and thermoesthesia demonstrated considerable individual variability during the cooling protocol (Figure 2). Water temperatures at vasoconstriction index, onset of shivering, and sustained shivering ranged from 31 to 16°C, 31 to 15°C, and 20 to 9°C, respectively. Vasoconstriction index (21.6°C, 95% confidence interval (CI): 18.5–24.7°C) and onset of shivering (20.5°C, 95% CI: 17.7–23.3°C) water temperatures did not differ significantly (*P* = 0.80); however, both values were significantly warmer than the water temperature at sustained shivering (14.6°C, 95% CI: 12.4–16.8°C; vasoconstriction index, *P* < 0.001; onset of shivering, *P* = 0.001). Similar differences were also present in the perception of cooling ratings (vasoconstriction index vs. onset of shivering, *P* = 0.43; vasoconstriction index vs. sustained shivering, *P* < 0.001; onset of shivering vs. sustained shivering, *P* = 0.002). Subjects reported the first incidence of shivering across the complete spectrum of perceived temperatures (“Neutral” to “Very Cold”) while sustained shivering occurred for all subjects between “Cold” and “Very Cold” temperature levels. Shiver onset and intensity also exhibited a range of temporal patterns across subjects in response to cold exposure (Figure 3).

### Relationship between skin temperature and thermoesthesia

**Figure 4:**
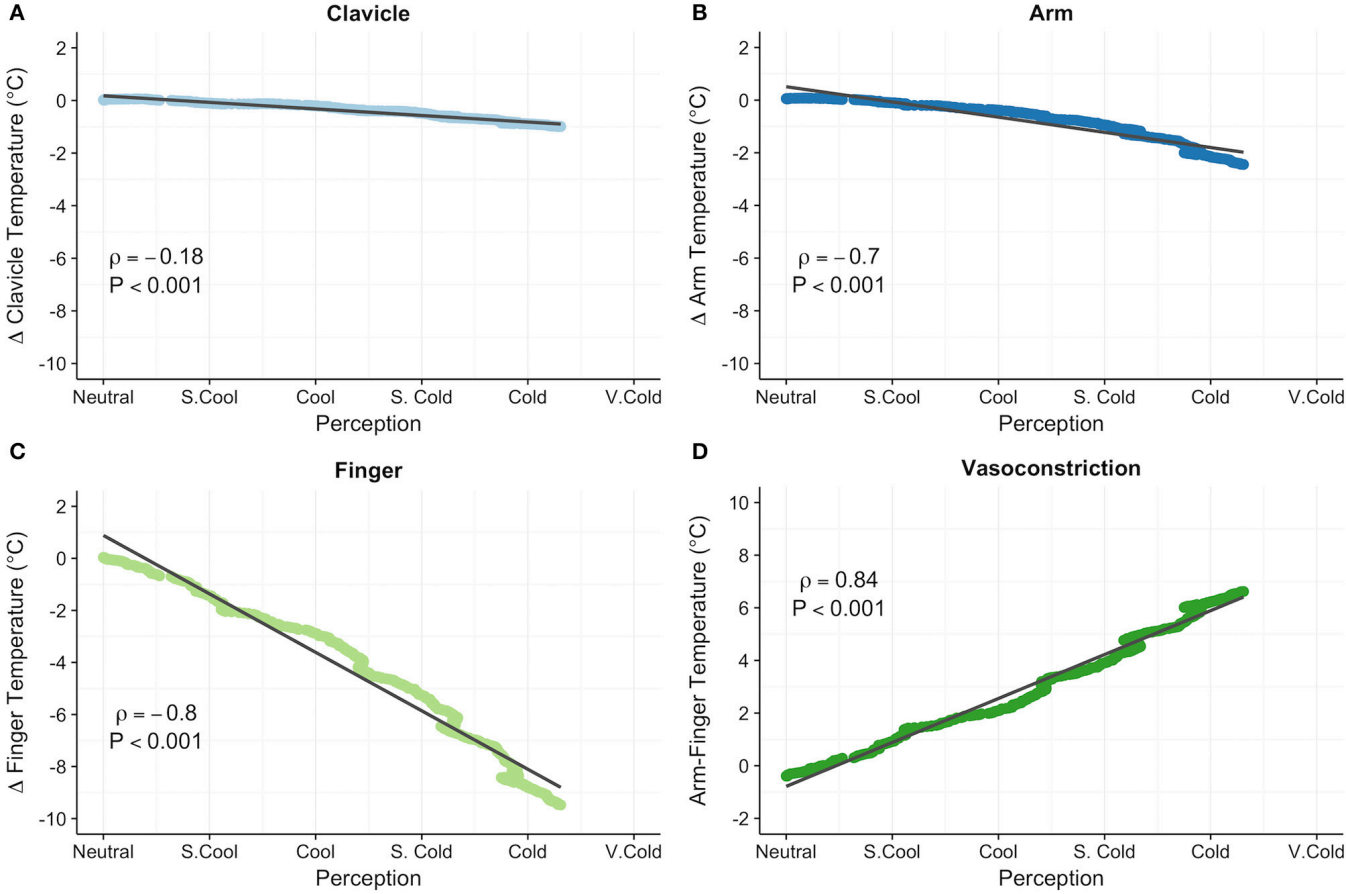
Clavicle (°C; **A**), forearm (°C; **B**), finger (°C; **C**), and peripheral vasoconstriction (forearm–finger, °C; **D**) skin temperatures correlated with subjective perception of cooling ratings. Relative clavicle, forearm, and finger skin temperatures are expressed as the change (Δ) in temperature from the end of the thermoneutral phase. Least squares best-fit lines are overlaid on the temperature data, which were linearly interpolated and averaged among participants. Small upward fluctuations in the temperature gradient (most notable in the finger skin temperature) were the result of two volunteers indicating a warmer perception rating when water temperature was adjusted (i.e., a brief stop of circulating cold water). Correlation (ρ) between temperature and perception was evaluated with the Spearman rank test.

Skin temperature data for 2 participants were excluded from analyses due to indications of pronounced peripheral vasoconstriction (>4°C arm to finger temperature gradient) at the end of the 15 min thermoneutral phase (Figure 2). Clavicle skin temperature demonstrated a weak relationship with thermoesthesia (ρ = −0.18, *P* < 0.001; Figure 4A). In addition, mean clavicle skin temperature decreased (−0.99°C, 95% CI: −1.7 to −0.25°C, *P* = 0.16) during the cooling protocol, but individual measures exhibited a wide range (−3.74° to 0.5°C) with warming of the region noted in 4 of the 10 subjects. As expected, arm (−2.4°C, 95% CI: −2.9 to −1.9°C, *P* = 0.002) and finger temperatures (−9.5 °C, 95% CI: −10.5 to −8.5°C, *P* = 0.002) decreased resulting in greater peripheral vasoconstriction (7.0°C, 95% CI: 6.2 to 7.8°C, *P* = 0.002) in response to cold exposure. Cold induced changes in skin temperature were also strongly related with perception of cooling ratings (forearm: ρ = −0.70, *P* < 0.001; finger: ρ = −0.80, *P* < 0.001; vasoconstriction: ρ = 0.84, *P* < 0.001; Figures 4B–D). Small upward fluctuations in arm, finger, and peripheral vasoconstriction temperature gradients (Figures 4B–D) were the result of two subjects indicating a warmer tGUI rating when the protocol water temperature adjustment caused a brief decrease in the circulation of cold water.

### Monitoring acute physiological responses to cold exposure

**Figure 5:**
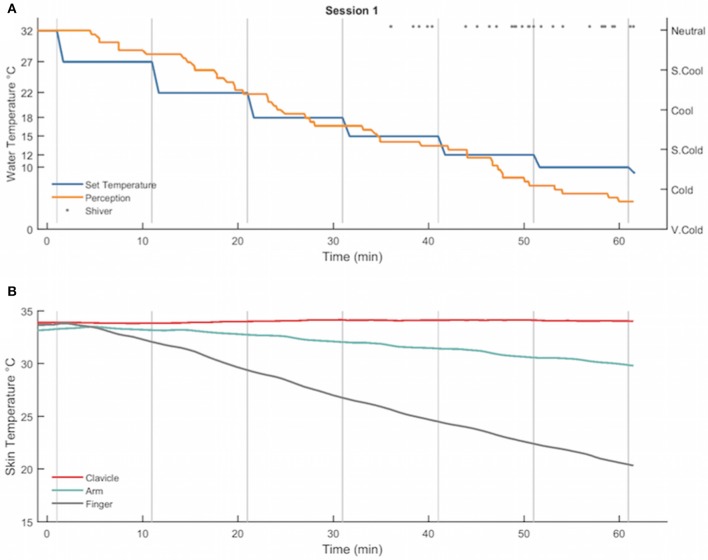
Example of the water temperature set points (°C, blue), subjective perception of cooling ratings (arbitrary units, orange), and self-reported shiver events (gray dots) **(A)** and clavicle (°C, red), forearm (°C, green line), and finger (°C, gray line) skin temperature gradients **(B)** recorded from a healthy, young male during a perception-based cooling protocol. During the protocol, the set point of the water temperature (blue line) was adjusted at the end of each 10-min cooling phase (vertical gray lines) according to the subject's thermal perception rating (orange line; see section Individualized, Perception-Based Cooling Protocol for details). The protocol terminated when the subject reported sustained shivering (>1 min in duration; gray dots).

Figure 5 displays the water temperature/perception of cooling profile (A) and absolute clavicle, forearm, and finger skin temperatures (B) recorded from a healthy, young male (age: 31 years, BMI: 24.0 kg/m^2^) during a perception-based cooling protocol. In this example, the participant indicated a gradual cooling sensation with sporadic shivering starting in the fourth cooling phase and progressing to a sustained shiver at the end of the sixth and final cooling phase (Figure 5A). In addition, the participant's clavicle skin temperature increased slightly, and arm and finger skin temperatures decreased in response to cooling (Figure 5B).

**Figure 6:**
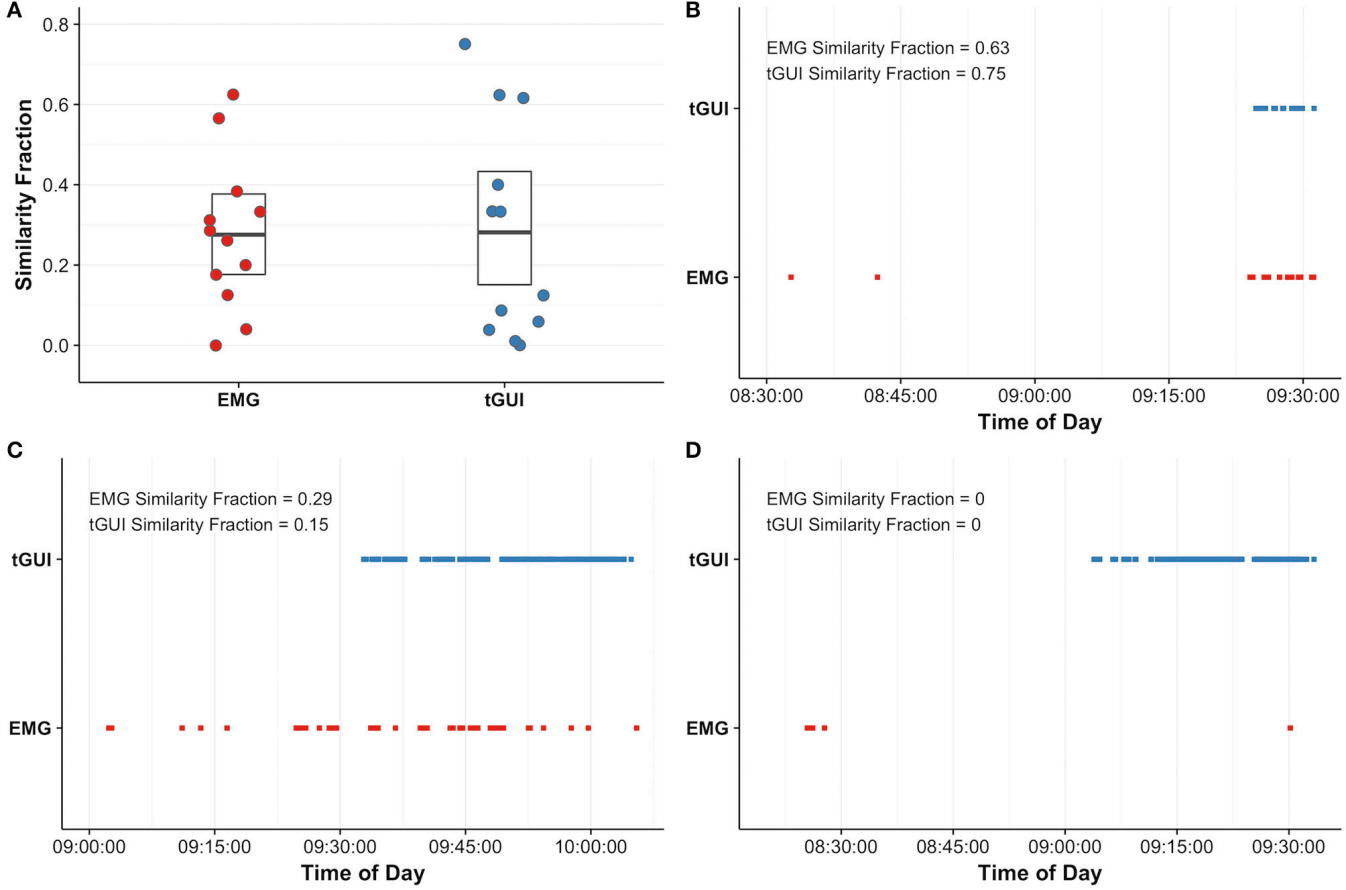
Similarity fractions (arbitrary units; see equations 2 and 3 for details) were calculated to evaluate the utility of surface electromyography (EMG; red) recordings of trapezius and sternocleidomastoid muscle activity and subjective events captured with the thermoesthesia graphical user interface (tGUI; blue) to quantify shivering **(A)**. The centerline in each box indicates the mean value, and the top and bottom of the box mark the 95% confidence intervals. Individual examples highlight good agreement between EMG and tGUI shiver events number and timing **(B)** and illustrate instances of poor specificity **(C)** and sensitivity **(D)** of EMG to measure whole body shivering. Brief shiver events may be difficult to visualize on plots **(B–D)** due to the resolution of the time scale.

For the total sample, EMG muscle activity and tGUI shiver events quantified shivering during the protocol; however, the number of events (EMG: 1–363; tGUI: 3–105) and similarity of shiver timing (i.e., similarity fraction; EMG: 0–0.63; tGUI: 0–0.75; Figure 6A) ranged between participants. Representative plots illustrate examples of strong (Figure 6B), moderate (Figure 6C), and poor (Figure 6D) similarity between EMG and tGUI shiver events. Notably, EMG activity in the moderate case often corresponded with movements not specific to shivering (researcher observations of deep breaths, speaking, or fidgeting) whereas TRAP and SCM muscles were not sensitive to shivering in the poor case example.

## Discussion

We describe a detailed methodology for an individualized, perception-based protocol to investigate human physiological responses to cooling. A small study was conducted to demonstrate the efficacy of our proposed method to individualize cooling, adapt cooling to the participant's cold sensitivity, and quantify physiological responses to cold exposure. Water temperature, perception of cooling, and shivering varied across participants, thus supporting the importance of personalized cooling protocols, such as the proposed method, for the study of cold-activated BAT (van der Lans et al., 2014). We also found a strong correlation between cold-induced changes in perception of cooling and peripheral vasoconstriction. Use of thermoesthesia ratings to guide adjustments in water temperature, therefore, may adapt cooling to the participant's cold sensitivity—an important consideration to replicate cold stress during longitudinal or cold acclimatization intervention studies of BAT physiology (Chen et al., 2016).

### Standardizing BAT activation: individualized, water-cooling paradigm

Individualized, water-cooling is necessary to apply a similar physiological cold stress and stimulate BAT consistently across participants (van der Lans et al., 2014). We examined water temperature and thermoesthesia with three distinct physiological markers associated with acute thermoregulatory responses to cold: vasoconstriction index, onset of shivering, and sustained shivering. When exposed to cold, decreases in skin temperature initiate vasoconstriction and shivering to offset heat loss and maintain body temperature (Castellani and Young, 2016). Vasoconstriction index, a 4°C arm-to-finger skin temperature differential, reflects a significant reduction in peripheral blood flow (Rubinstein and Sessler, 1990). Specific shivering benchmarks, however, are less clearly defined because the onset, extent, and intensity of shivering differ between individuals (Tikuisis et al., 1991; Castellani and Young, 2016). Using individualized cooling, we found no significant difference in vasoconstriction index and onset of shivering mean water temperature and thermoesthesia. While this finding may suggest that both physiological markers represent a similar cold stimulus, we noted considerable individual variability in the pattern of shivering, which may reflect differing contributions of skeletal muscle contractions and BAT to cold-induced thermogenesis (Blondin et al., 2017). Because heat production increases as a function of cold exposure (Blondin et al., 2015), continuing to apply a cold stimulus until the participant reports sustained shivering may increase the likelihood of maximizing BAT activity across subjects. Quantifying the extent of BAT activity and its relationship with cold exposure, however, remains a critical area of future research.

### Advantages of perception-based cooling gradients

Previous cooling methods employ fixed temperatures or predetermined temperature gradients across participants regardless of differences in sex, age, body composition, and cold tolerance (Ouellet et al., 2012; Muzik et al., 2013; van der Lans et al., 2013; Bakker et al., 2014; Chondronikola et al., 2016; Gifford et al., 2016; Martinez-Tellez et al., 2017b). However, individual characteristics and external factors, such as prior cold exposure or exertional fatigue, contribute to variability in the thermoeffector responses to cold and BAT activity (van der Lans et al., 2014; Castellani and Young, 2016) and therefore the rate at which individuals lose heat and defend against heat loss. For example, overweight and obese participants have been shown to preserve body temperature (Gifford et al., 2016) and delay the onset of shivering (Vijgen et al., 2011) during cold exposure. Perception-based cooling may offer an approach to account for this variability. Thermoesthesia correlated strongly with peripheral vasoconstriction, an independent physiological measure of cold sensitivity (Castellani and Young, 2016). This association suggests the perception-based cooling protocol may improve the sensitivity of detecting personalized, physiological cold stress thresholds in a heterogeneous group of research participants. Further, adapting cooling gradients to cold sensitivity may be advantageous to explore the effects of cold acclimatization on BAT. Thermoregulatory physiology (blunted cutaneous vasoconstriction, Castellani and Young, 2016) and BAT activity (van der Lans et al., 2013; Blondin et al., 2014; Hanssen et al., 2015) adapt to cold habituation. Perception-based cooling, therefore, may enable the level of cold stress to be replicated within an individual before and after an intervention. A comparable cold stress will improve our understanding of any differences in the timing and magnitude of BAT heat production, and its potential role in whole-body energy metabolism and weight maintenance.

### Quantifying acute physiological responses to cold exposure

#### Skin temperature estimates of peripheral vasoconstriction and BAT activity

Changes in supraclavicular skin temperature have been proposed as a surrogate measure of BAT activity (Boon et al., 2014; Chondronikola et al., 2016; van der Lans et al., 2016). Although biopsy or medical imaging (see review of BAT imaging techniques, Sampath et al., 2016) are required to confirm the presence of BAT, increased clavicle skin temperature suggests the presence and activation of BAT in a small subset of volunteers in this study. Maintenance or slight decreases in supraclavicular skin temperature may also indicate active BAT (Chondronikola et al., 2016; van der Lans et al., 2016), but a predictive relation between the magnitude of the skin temperature change and BAT volume remains unclear. Nevertheless, supraclavicular skin temperature adds value to the cooling protocol as an indirect approach to screen participants for potential BAT depots prior to conducting more invasive or expensive medical imaging studies.

Peripheral skin temperatures provide a method to monitor the efficacy of the cooling protocol. Upon cooling, skin temperature decreases as vasoconstriction reduces skin blood flow (Castellani and Young, 2016). Therefore, we advise monitoring finger skin temperature to ensure the participant experiences cooling. We further propose using the arm-to-finger temperature gradient to confirm the thermoneutral temperature (Chen et al., 2016). In this study, skin temperature gradients indicated significant vasoconstriction (>4°C, Rubinstein and Sessler, 1990) in 2 of the 12 volunteers following 15 min at a nominally thermoneutral temperature (self-reported “Neutral” on the tGUI tool). Future work should consider warming the blanket water temperature during the thermoneutral phase until the arm-to-finger gradient is zero to reduce the effect of inadvertent cold exposure on experimental outcomes. Although pronounced changes in core body temperature are unlikely with mild cold exposure (Castellani and Young, 2016), measurement of core body temperature and additional skin temperature sites can be included to assess thermoeffector responses to the cooling protocol more broadly if desired (Martinez-Tellez et al., 2017a).

#### Utility of the thermoesthesia graphical user interface (tGUI)

The tGUI tool creates a simple interface for participants to translate perception of cooling and shivering into a formal rating system. Subjective ratings integrate the complex and often imperceptible physiological and mental responses to an event into a relatable format that encourages standardized evaluation (Hart and Staveland, 1988). Although thermal sensation ratings have been incorporated into previous studies (Muzik et al., 2013; van der Lans et al., 2013; Matsuda-Nakamura et al., 2015; Chondronikola et al., 2016), fixed evaluation time intervals or use of verbal questioning limit event recall and may result in qualitative differences between participants (Hart and Staveland, 1988). With the tGUI tool, participants can interact with a keypad to adjust the thermoesthesia scale instantaneously with minimal muscle movements or need for verbal cues. Instruction and training (e.g., familiarization session), however, are necessary to help participants learn to deliberately categorize temperature sensations or shiver events in a consistent manner within the context of the cooling protocol environment.

#### Comparison of self-reported and EMG shiver events

Self-report of shiver events with the tGUI tool also provides an alternative to surface EMG to monitor shivering during the cooling protocol. While surface EMG is widely used to measure muscle activity, electrical signals result from both shivering and voluntary muscle movements. Equipment constraints further limit the utility of EMG to record shivering in only a select number of superficial muscles. Shivering, however, often originates in the deep muscles of the torso (Bell et al., 1992) and exhibits unique onset, intensity, and muscle recruitment patterns in each individual (Tikuisis et al., 1991; Castellani and Young, 2016). In this study, participants reported the start and end of any involuntary muscle movement in real-time with the tGUI tool resulting in a more specific and sensitive measure of whole-body shiver onset and duration compared with surface EMG in some individuals. Moreover, the tGUI keypad and visual display operate within the constraints of medical imaging environments that complicate use of most EMG systems (e.g., challenges viewing participant movements, ferromagnetic cables, and electrical noise). The tGUI shiver events, however, are susceptible to self-report errors (e.g., incorrect button press or failure to identify a muscle movement) and lack resolution and intensity information required to assess skeletal muscle metabolism. Careful selection and increased sampling of EMG muscle activity or use of more quantitative methods should be considered if whole-body muscle metabolism is a desired study outcome (Blondin et al., 2015).

### Limitations

Performing the perception-based cooling protocol in the context of a medical imaging environment (Sampath et al., 2016) is a logical next step to determine how the presence and activation of BAT contributes to the physiological responses to cold exposure measured in this study. In addition, testing the protocol in populations with greater diversity in age and body composition than tested here would increase its applicability in future studies. Older participants experience a lower threshold for peripheral vasoconstriction and onset of shivering (Frank et al., 2000), but it is unknown if these physiological adaptations would adversely affect the ability to use thermal perception to personalize cooling. Additional cooling blankets may also be needed to ensure the protocol applies a similar cold stress to individuals with increased body surface area or subcutaneous adipose tissue. It should also be appreciated that the perception-based cooling protocol may not be appropriate for all individuals. Future studies are needed to determine if medications known to affect thermoregulation and BAT activity (Cuddy, 2004; Mukherjee et al., 2016) or chronic conditions, such as diabetic neuropathy (Gilmore et al., 1990), affect the ability to individualize, adapt, and quantify cooling in populations with varying therapeutic treatments or pathological conditions. Finally, a repeatability study should be conducted to evaluate the consistency of an individual's shiver threshold for experiments requiring cold exposure on multiple days (Martinez-Tellez et al., 2017b). Strict enforcement of pretest restrictions (section General Participant Preparation Guidelines) may control for some variability within the individual, but it is unknown how fluctuations in acute cold exposure or exertional fatigue (Castellani and Young, 2016)—factors difficult to control and measure during an experiment—alter cold sensitivity and/or the threshold for shivering.

## Summary

Building upon current best practices, we developed an individualized, perception-based protocol to investigate human physiological responses to cooling. A small feasibility study demonstrated the ability of the protocol to individualize, adapt, and quantify cooling in a cohort of healthy adults. Future implementations of the protocol should assess its efficacy in diverse populations, in combination with cold acclimatization or pharmacological intervention, and in medical imaging environments. As some of the underlying assumptions regarding BAT physiology are better understood, we hope the methods described here provide a foundation to create consistent cooling protocols to improve inter-study comparisons of experimental outcomes.

## Reproducible research

Data and code are available for public download from a version-controlled repository (https://github.com/ccoolbaugh/FrontPhysiol_Coolbaugh_Cooling_Protocol) to reproduce the figures and statistical analyses in this article (Coolbaugh, 2018). Example data recorded during a perception-based cooling protocol are accessible and can be analyzed with the individualized cooling data analysis summary code (Coolbaugh and Bush, 2017).

## Ethics statement

This study was carried out in accordance with the recommendations of the Vanderbilt University Medical Center Institutional Review Board with written informed consent from all subjects. All subjects gave written informed consent in accordance with the Declaration of Helsinki. The protocol was approved by the Vanderbilt University Medical Center Institutional Review Board.

## Author contributions

CC, EB, EW, and TT conception and design of research; CC, EB, and EG performed experiments; CC and EB analyzed data; CC, EB, EW, and TT interpreted results of experiments; CC drafted the manuscript; and CC, EB, EG, EW, and TT edited and revised the manuscript. All authors approved the final version of the manuscript and agree to be accountable for the content of the work.

### Conflict of interest statement

The authors declare that the research was conducted in the absence of any commercial or financial relationships that could be construed as a potential conflict of interest.
